# Complement Fixation Tests as Applied to Dental Practice

**Published:** 1919-06

**Authors:** Angelo Zabriskie

**Affiliations:** Ridgewood


### COMPLEMENT FIXATION TESTS AS APPLIED TO DENTAL PRACTICE

BY ANGELO ZABRISKIE, D.D.S., RIDGEWOOD

Should all dead teeth be extracted?

Should dead teeth be treated to eliminate systemic infections?

To what extent is the system affected by infections in and about the mouth?

These three questions have been given great prominence in dental and medical circles recently and are leading us to new findings regarding focal infection and the part we hold as mouth specialists in guarding human health and life.

I want to say something about a method of procedure when a patient is referred to the dentist by the physician after he has treated the case. He may say, "The cause of your ailment is your teeth. I have treated you for other things and get no result.'' This is the way we so often have these cases referred to us, and now what are we going to do with them? The first thing, of course, is a thorough examination of the tissues of the mouth. Are they healthy and are the teeth in good condition? Does the patient breath properly? What are the systemic symptoms: obscure pains, gastro-intestinal disturbances, or what? Has a full blood examination been made, has a urine analysis been made, and what did they show? The next step is a full radiographic dental series and, in addition, the accessory sinuses should be radiographed. May I say here that I do not believe our radiographs lie, as so many men claim, but that our technique or our ability to interpret our X-ray findings is at fault? We find from our dental series that there are rarefied areas of bone, canals not properly filled, pyorrhea pockets that were unsuspected when our preliminary examination was made, distinct abscessed areas or larger areas of necrosis, bone changes that are not normal, cystic formations and a great many other things of abnormal appearance that the film shows.

The patient has been sent to us, remember, for a systemic condition that may be obscure or perhaps a well-defined case of arthritis, heart disease of one or more of the different forms, neuritis, kidney, gastro-intestinal or almost any disease that the human body is heir to.

Can we now say to the patient that we have found infection in the mouth and that it should be eliminated and will effect a cure? This is what the patient insists upon knowing before giving consent to the elimination of the suspected focus. Is he going to have apparently a good tooth or teeth removed when they have never caused pain and have always been very serviceable? We show the radiographs and the areas of suspected trouble, but even this is not convincing, because he knows of another person who followed the advice of the removal of teeth or the opening of a sinus and derived no benefit whatever. Must our answer be that the radiographs show infection and that on general principles it should be eliminated and that after two or three months, if the systemic symptoms are still present, it would be well to look elsewhere for the trouble? We might also say that if the teeth are involved the worst ones could be removed and have the rest treated or have them all treated, and then if the systemic symptoms failed to disappear, have them extracted. Or, if the radiographs showed a satisfactory clearing up of the infection after treatment, a regeneration of bone, a diminution in the size of the granuloma or a perfectly filled root canal, that the systemic condition is surely from another cause and can not come from the teeth. All of this is very unsatisfactory. We know it, and some patients will find it out sooner or later.

Now we must consider the effect of the foci of infection that we have just been talking about on the general system. Whether the infection comes from the teeth, accessory sinuses, tonsils or what not, it makes very little difference. The effect remains practically the same.

The blood and lymphatic circulations we believe are the carriers of the causes of systemic disturbances. The bacteria themselves are formed in the focus and here they grow, reproduce themselves, assimilate food in order to live and excrete something we term toxins. These toxins are taken up by the blood stream and immediately the natural forces of nature are called into play to counteract or neutralize these toxins. This is very satisfactorily done for a time. The time may be short or may extend over a period of years. This we can not tell, as it depends upon the virulence of the bacteria, the condition of the patient, his mode of living, his diet, the amount of exercise and sleep and various other things too numerous to mention, and a good many causes that I do not believe we are aware of at present.

The toxins, but not the bacteria, are present in the blood stream. If the bacteria were coursing through the circulation and propagating there we would have a true bacterima and death would ensue within hours instead of weeks or years, as it does in toxemia. Therefore, we have only toxemia to deal with in the majority of our cases. If there are other foci of infection in the body we will still have toxemia, but the toxin may be of a different nature, according to the different nature of the bacteria. For instance, one may find tooth infection that produces streptococcus and a venereal infection that produces gonococcus. While these germs are not in the blood stream their toxins are. Hence we have toxemia and so-called infection.

Let us, for example, take a case of tuberculosis and tooth infection together, where we know that the resultant toxemia is due to these bacteria. The case is being treated for pulmonary tuberculosis and the tooth infection is ignored. Something is being missed, something is not being done. When diphtheria bacillus is grown alone in the laboratory it grows just so fast, and when streptococcus grows alone the growth we will say is normal, but when diphtheria bacillus and streptococcus are combined the growth is increased above normal. Therefore tuberculosis and strep, gonococcus and strep, or several foci of different bacteria being present in an individual at the same time may be a big factor in the diagnosis, treatment and prognosis of disease. I think that when we have some other infection of the body, and if there is a streptococcus infection accompanying it, whether it be from teeth, tonsils, accessory sinuses or elsewhere, our prognosis will not be as favorable and the case will not clear up as it would if the strep were removed as early as possible, or if it were not present at all.

We know of the high death rate in the recent epidemic of influenza and the resultant pneumonia. In these cases of septic pneumonia, streptococcus is found. Have these patients a focus of strep somewhere in their bodies? Is it around the teeth, possibly? And would the great number of deaths have occurred and would the pneumonia have developed a septic type if the patients had not had this focus of strep that has been present, possibly for years, and never caused any serious disturbances? There was only a toxemia that the system was successfully combating until the acute infection of influenza manifested itself. The general tone of the system being lowered, the strep helped the other bacteria and the other bacteria helped the strep, and death ensued in several days.

I think that the preceding has answered in a degree how the system may be affected by infection in and about the mouth.

We are still groping around in the dark, asking questions and hoping against hope that when we eliminate the suspected infection by extracting or treating suspected teeth, removing suspected tonsils or treating the accessory sinuses, that our systemic symptoms will disappear. If we obtain a good result we are lucky, if some improvement is accomplished we are almost as lucky, but if no good is felt by the patient we will certainly be condemned and the patient is discouraged.

There is a method of examining the blood that will help us to clear the question of the cause of the patient's condition. We must keep in mind to use all other means of diagnosis at the same time. You are all no doubt familiar with the Wasserman test for syphilis. There is also a similar test made for typhoid and similar tests made for other suspected infections. The complement fixation test is the one of which I speak. This test as generally accepted was not very satisfactory and could not be depended upon, but I have reference to a modification which is reasonably reliable as a help in diagnosis.

When a patient is sent to me by the physician, suffering from some systemic disturbances, it is because the teeth are suspected. The case has probably been in the hands of one or more physicians and as a general rule is obscure or a stubborn one that does not respond to treatment and does not clear up readily.

First, I make an examination of the mouth, teeth and accessory sinuses both radiographically and otherwise. Then I insist upon a full blood examination.

Be it granted that I find suspected infection that comes under my jurisdiction. Can I say that this must be removed or treated and that a cure will result? Yes, if I have taken the blood, six or eight cubic centimeters, from the median bacillic vein of the arm. My blood examination will give me a Wasserman, complement fixation for colon bacillus, gonococcus, streptococcus, tuberculosis, staphlococcus, catarrhalis, etc. It also gives me a blood culture and blood count which is taken in the regular manner. With all this data I have something to work with and can give a diagnosis or information leading to a diagnosis.

Case A—Female, suffering from arthritis deformans. Confined to bed for over one and a half years. Radiographs showed tooth infection. The Wasserman was negative, the complement fixation gave a four plus streptococcus and a three plus gonococcus. Just think what it would have meant to extract the teeth and obtain no result, no improvement. That is just exactly what I would have done if I had not taken the blood. Later another practitioner might have hit upon the remaining cause of the trouble, and then, after treatment, the case would have cleared up. The dentist would be severely criticised for doing an unnecessary operation, when in reality a part of the cure by elimination of the foci of infection was due to the elimination of thé strep infection, and consequently the elimination of its toxins from the blood.

Case B—Male, 38 years old; for four years had been in poor health. During this time he had several attacks of boils on his back and neck. He had been from one physician to another and had used every means of diagnosis that presented itself. The urine analysis showed a very small percentage of sugar, and finally he was informed that he had diabetes. The teeth were suspected as a possible cause. The condition of the patient was so grave that I am sure any one of us would have extracted any or all of the teeth on general principles. The radiographic examination of the teeth was negative. The blood showed that there was a positive colon bacillus reaction when a complement fixation was made. Wasserman and every other test was negative. The fact was established that there was no strep infection. The teeth were eliminated. The radiographic gastro-intestinal series showed a complete gastro enteroptosis. A floating kidney was found and stone in the bladder was later demonstrated. Just think of removing teeth in this case with or without radiographs.

Case C—Female, about 28 years old; had been ill for six or seven years. The teeth had been suspected from the first. The patient had been under almost constant medical and dental treatment for the entire period of her illness. Then the appendix was removed. The old symptoms of hysteria, neuritis and neuralgic pains still prevailed. Impacted third molars were removed from the lower jaw, and finally a mental condition was decided upon as the probable cause. When I saw the case and had a complement fixation test made the Wasserman was four plus, gonococcus three plus, streptococcus four plus and two plus staphlococcus and catarrhelis was demonstrated. The case was sent to me for dental treatment. The treatment or extraction of the infected teeth would not have effected a cure. But the extraction of the infected teeth, curretment of the infected areas and the elimination of all dental infection was certainly a part of the treatment.

Case D—Male, about 30 years old; had a severe heart affection. The patient had been a Marathon runner and was at the time a trainer in one of our largest universities. The case was first diagnosed as a condition resulting from overexercise. A complement fixation test showed a streptococcus reaction. The other tests were negative. The dental radiographic series showed infection. A negative complement fixation was obtained within forty-eight hours after the elimination of the focus of infection. The patient fully recovered and shortly afterward passed an examination for life insurance. Later on, when war was declared, he was accepted in the aviation corps. But during the final tests for "off the ground" service he had a recurrence of his former symptoms. There was found to be a return of the streptococcus infection. The tonsils were found to be diseased and were removed. The patient fully recovered and was accepted for flying.

Case E —Male, 74 years old: suffering from a heart affection, gastro-intestinal disturbances and a general condition of old age. The blood showed a streptococcus reaction to complement fixation, other tests being negative. The radiographs showed infection about the teeth. Upon the removal of the focus of infection the blood showed a negative reaction to streptococcus in twenty-four hours. This was a complicated case that also showed a gastro-enteroptosis. The stomach and intestines were down below their normal position in the abdomen. After treatment for this condition improvement was very marked.

Here I want to give rise to another thought. Was the tooth infection primary and did the toxemia resulting so lower the general tone of the whole system that the gastro-intestinal condition was the result? Or was the toxemia, due to the gastro-intestinal condition, primary, and did this so lower the resistance of the tissues around the devital teeth that they become readily open to infection?

In conclusion, can we safely treat infected teeth to eliminate systemic infection? Yes, providing that after treatment we can show a negative complement fixation of the blood after first having demonstrated that the infection was originally from this source. Perfectly filled root canals are not immune to infection, and if any dead tooth is present where there is systemic infection it must be regarded with suspicion. There is no reason why root canals can not be treated, why teeth can not be devitalized, providing they will never cause infection. If infected teeth are treated with a view to eliminating infection we must be able to show that the toxemia, if it is in the blood, has been eliminated and that no toxemia will be a result thereof. —New Jersey *Dental Journal.*

